# Understanding the vaccine hesitancy of COVID-19 in Benin

**DOI:** 10.1371/journal.pgph.0004267

**Published:** 2025-02-25

**Authors:** Daleb Abdoulaye Alfa, Jean-Yves Le Hesran, Inès Boko, Anani Agossou, Aurore Atchadé, Marc Fiogbé, Emmanuel Yovo, Sandrine Hounsa, Achille Massougbodji, Gilles Cottrell

**Affiliations:** 1 Institut de Recherche Clinique du Bénin/ IRCB, Abomey-Calavi, Bénin; 2 Institut de Recherche pour le Developpement (IRD), MERIT Université Paris Cité, Paris, France; University of Oxford, UNITED KINGDOM OF GREAT BRITAIN AND NORTHERN IRELAND

## Abstract

Vaccination campaigns against COVID-19 have been set up in all countries. The aim was to reach a sufficient vaccination threshold to ensure herd immunity. In Benin, the objective was to achieve 60% coverage. However, coverage was only 35% in May 2022. People were reluctant to be vaccinated. We had set up a population-based study to investigate these barriers to vaccination. Our approach was qualitative (80 semi-structured interviews with vaccinated and non-vaccinated people) and quantitative (179 questionnaires with CHWs (Community Health Workers) in urban and rural areas. To analyse the qualitative data, thematic sorting was carried out, while the statistical analysis of the data was carried out using SPSS and Excel software. Perceptions and concerns about COVID-19 revealed widespread mistrust of the disease and vaccination. Part of the population doubted the existence or seriousness of the disease, with over 70% of CHWs reporting that people did not perceive the reality of the disease in their daily lives. These doubts were reinforced by the limited impact of the disease and political interpretations of the pandemic, often viewed as a tool for state control. Mistrust of vaccines was even more pronounced, with over 90% of CHWs indicating that people were concerned about the novelty of vaccines and doubts their effectiveness. Rumours circulating on social networks amplified these concerns, fuelling fears about vaccine safety. Fear of stigmatisation, forced isolation and the impossibility of carrying out traditional funeral rituals heightened people’s reluctance. The requirement to sign a consent form absolving the state of responsibility for side-effects further deepened these suspicions. Our study confirmed a strong reluctance to vaccinate against COVID-19. It highlighted the critical role of media and social networks and the necessity for authorities to address these factors in communication diseases to ensure efficient disease control.

## Introduction

Following the discovery of the COVID-19 virus in China and its rapid spread throughout the world, in March 2020 the WHO declared a state of pandemic [1]. Initially, governments put in place response measures, including border barriers and a period of population confinement [2–4]. At the end of 2020, private pharmaceutical laboratories offered the first vaccines against COVID-19 and, under the aegis of the WHO, mass vaccination campaigns were set up in all countries [5]. The aim was to reach a sufficient vaccination threshold to achieve herd immunity [6]. However, in all countries, the campaigns struggled to achieve their objectives [7–9]. Anti-vaccination currents developed, particularly on social networks, and many members of the public were reluctant to be vaccinated because they felt that the vaccine was still too new to be able to assess its effectiveness and, above all, its danger. This may have been due to vaccine hesitancy [10–12]. The WHO defined vaccine hesitancy as a “delay in acceptance or refusal of vaccines despite the availability of vaccination services”[13]. It was a process that ranged from complete acceptance to complete rejection [14–16].

The government of Benin was affected by the COVID-19 pandemic on 16 March 2022. By April 2021, the toll had risen to 161 deaths and 25,552 positive cases [17]. A strong resurgence of cases was observed, with a peak of 4,500 cases in September 2021 [18,19]. The departments of Littoral, Atlantique (the site of our study) and Ouémé were the most affected, while the others had less than 10% of cases [20]. A number of countermeasures were taken by the government, including - recommending vaccination to anyone over the age of 18, including teaching and administrative staff in public and private establishments before the start of the new school year, students, military and paramilitary personnel and motorbike taxis entering or leaving the country, suspending the participation of any public official or private sector employee who had not been vaccinated against COVID-19 in meetings (meetings, workshops, forums, seminars, etc.) on national territory [17].

The Ministry of Health had set for itself the target of having 60% of the population vaccinated. This coverage rate was a recommendation from the national office of the World Health Organisation [20]. By March 2022, only 35% of the population had received the first injection. It should however be pointed out that basic immunisation coverage for children aged 12-23 months was 57% on average for Benin and 60% in the study area, according to the Demographic Health Survey [21].

Like several other African countries, Benin experiencing low uptake of vaccination against COVID-19 [22–26]. Located in West Africa, it is bordered to the east by Togo, to the west by Nigeria, to the north by Burkina Faso and Niger, and to the south by the Bay of Benin in the Gulf of Guinea. The country is divided into 12 administrative departments, French is the official language, but each ethnic group has its own language, which is also spoken. Benin’s national health system is organized around zone hospitals in major cities such as Cotonou, Porto-Novo, Parakou, Abomey, Ouidah and Natitingou, and health centers in neighborhoods, secondary towns and rural and semi-rural areas.

The Theoretical Mean Action Radius (RMAT) is used to estimate the average distance separating populations from a health unit. The average theoretical radius of action was 3 km in the Abomey-Calavi-So-Ava zone. In the Zè-Allada-Toffo health zone, the average theoretical radius was 3.6 km [27]. Financial aid from international organisations is helping to make up for the shortage of medical staff and medicines.

To encourage people to get vaccinated, the Ministry of Health broadcast awareness-raising messages on television and radio [28]; On the other hand, certain coercive measures have been taken, such as compulsory vaccination for certain professions (police officers, teachers, medical and paramedical staff, pharmacists, nursing assistants, administrative staff of public and private health facilities, staff of pharmaceutical pharmacies), banning access to public buildings for people who have not been vaccinated, and compulsory Vaccination Passes for sick guards before they can access the health centre [29]. However, nothing has been done and we are still a long way from achieving our targets. In order to achieve a sufficient vaccination rate and significant protection of the population [30], it would have been necessary to prepare a communication that took into account all the questions that the population was asking [8].

This is why we wanted to document the reasons for vaccine hesitancy, we chose two entry points and interviewed CHW. CHW play an essential role in improving the health of the population. They are responsible for a number of activities, including treating cases of uncomplicated malaria and respiratory infections, raising awareness of health issues within the community, home visits and, finally, screening and referral of malnourished children under the age of five. The CHW come from the community (the people whose health problems they are responsible for). They were chosen because they act as an interface between the local population and the health workers in the dispensaries [30]. In the fight against COVID-19, their role was to inform people about the government’s response policy and, to raise awareness of vaccination. They therefore seemed to be very well placed to report people’s concerns, and to share with us their perception of how their intervention and the government’s measures were received by the population. We also took an interest in the general urban population by conducting semi-directed interviews in Abomey-Calavi (a suburb of Cotonou). The aim was to gain a better understanding of the mechanisms by which people’s attitudes to COVID-19 vaccines and government measures are constructed. This will enable the government to adjust its response measures and prepare better for future epidemics.

## Methods

### Ethics statement

This study was approved by the Comité National d’Ethique de la Recherche en Santé du Bénin (CNERS ethical authorisation reference: N°129/MS/DFRMT/CNERS/SA). All participants gave written consent before being interviewed. Our study did not involve experiments on humans and/or the use of human tissue samples.

### Data collection method

To carry out this study, we chose a dual methodological approach: qualitative, with individual interviews and focus groups, and quantitative, with the administration of a questionnaire to CHW on their own perception of COVID-19 and on people’s concerns about COVID-19. These two methodological approaches are complementary. The qualitative approach can be used to highlight new decision-making logics and processes, or new relationships between the phenomena under study [31,32]; it can also be used to develop new hypotheses. The quantitative approach, on the other hand, is better placed to test, validate or justify hypotheses [31–34].

We chose the commune of Zè for two reasons. Firstly because of its rural character, and secondly because it is one of the communes in the health zone where the COVID-19 patient care center is located. The commune of Abomey-Calavi was chosen because of its peri-urban character and the request of the health authorities to understand the strong reluctance of the population to be vaccinated against COVID-19 in this commune.

### Qualitative approach

In the Abomey-Calavi commune, 80 individual community interviews were carried out, 60 with non-vaccinated people and 20 with vaccinated people. To identify the subjects to be interviewed, we used the snowball technique [35] which is a non-probability sampling method whereby the first subjects in the study recruit other subjects from their entourage. This snowball technique was used because it was a difficult target to identify in the community[36]. To ensure variability, we have made a well-considered choice [37] which takes account of religion, profession and age. Reasoned choice gives the auditor sufficient flexibility to use his or her professional opinion to select the elements that seem most informative.

All interviews were recorded and transcribed in full. They were first translated into French when they had been conducted in the local language. Based on the themes in the interview guide, a thematic sorting of all the transcribed interviews was carried out. Emerging themes were taken into account in the analysis [38–40]. All the information was analysed by category of meaning, a content analysis was then carried out.

Focus groups were also held with CHW in the communes of Zè and Abomey Calavi to gain a better understanding of the issue through participant interaction. [41,42]. The CHW report to the national public health directorate. They are the vanguard of the community health strategy in Benin, on which the health authorities have also based their strategy. There are two types of CHW. Some deal with the ‘full package’ (health promotion and first aid, including malaria). They work in the more remote villages (more than 5 km from the health center) and cover 80 households, or an average of 400 people. The ‘promotional package’ deal solely with health promotion activities (awareness-raising). They operate in a radius of less than 5 km around the health center. They are responsible for around 120 households, or an average of 600 people. They are not responsible for providing first aid, so as not to compete with the health centers. These two types of CHW have been involved in awareness-raising activities to prevent COVID-19.

### Quantitative approach

The Abomey-Calavi commune where we worked had 105 CHW, there were 84 in the town of Zè. We were able to pass a questionnaire to 100 CHW in Abomey-Calavi and 79 CHW in Zè. The main objective of this questionnaire was to obtain feedback on the population’s perception of COVID-19 and the vaccine, as well as on the government’s decisions through CHW. CHW are an interface between health workers health centers and the population. They live in the community and share with people their daily experiences of health problems. We wanted them to share with us the information and perceptions that were circulating in the community about the COVID epidemic.

Through this questionnaire, several themes were addressed: perception of the vaccine by CHW, perception of COVID-19 and the vaccine by the population, response measure taken by the government, accessibility of vaccination for the population, vaccination against COVID-19 in children, confidence in the health authorities regarding vaccine insurance.

The questionnaires were administered in French and in Fon for those who were not comfortable speaking French. The questionnaires were administered to the CHW outside the health center to give them freedom of expression outside the influence of the health workers who are their direct supervisors in the commune.

We used digitised questionnaires on the KoboCollect platform, with automatic checks for skip patterns, consistency and completeness of key information. This approach to data collection had the advantage of allowing an initial level of quality control of the data collected. The data was saved on the KoboCollect server.

Statistical analysis of the data was carried out using SPSS and Excel. We used the Chi² test to compare the characteristics of the CHW population between the two communes in terms of sex, level of education, employment status, method of remuneration and time spent with the population in Table 1 and between the vaccine and non-vaccine population in Table 2 (age, gender, religion). Statistical tests are given at 5% alpha risk (p=0.05).

**Table 1:** Socio-demographic characteristics of CHW in the 2 communes.

		Abomey-Calavi (N)	%	Zè (N)	%	P
Gender	Men	38	38	79	100	P < 0.001
	Female	62	62	0	0	
Education	Primary	10	10	22	28	P < 0.001
	Secondary	70	70	55	70	
	University	20	20	2	3	
Relay status	Full package	75	75	75	95	P < 0.001
	Promotional package	25	25	4	5	
Salary Status	NGO	80	80	64	81	P < 0,04
	Ministry of health	13	13	15	19	
	Volunteer	7	7	0	0	
Time spent	0-20%	19	19	13	16	P = 0.13
	20-50%	37	37	42	53	
	50-70%	35	35	21	27	
	70-100%	9	9	3	4	

**Table 2:** Socio-demographic characteristics of people interviewed in the population.

		Vaccinated people	Unvaccinated people	
Age	18-30	8	22	P = 0.32
	30-45	5	19	
	45-60	7	19	
Gender	Men	10	28	P = 0.07
	Female	10	32	
Religion	Musulman	8	15	P = 0.43
	Catholic	7	25	
	Protestant	5	20	

## Results

### Presentation of the groups interviewed

For the quantitative component, we interviewed a total of 179 CHW, 100 in Abomey-Calavi and 79 in Zè. The CHW were all men in Zè, whereas we had a majority of women in Calavi (62%) (Table 1).

These results show that the profile of CHW differed between the two zones, except for the percentage of the time devoted to their work with the population. In particular, almost all CHW in Zé were men, and all had full package contracts. 1/5 of CHW in Abomey-Calavi had a university degree, compared with 3% in Zé, which can be explained by the proximity of Abomey-Calavi university. Many of those recruited on promotional package were young university graduates looking for work. Almost all the CHW in Zè (95%) were part of the full package (caregiver task and awareness). In Calavi, 25% of the CHW we met only did awareness (promotional package).

For the qualitative component, we carried out focus group with CHW. 165 of the 179 CHW who responded to the questionnaire took part in 10 focus groups, 90 in Abomey-Calavi and 75 in Zè. We carried out interviews in general population in Calavi., 60 with non-vaccinated people, 32 women and 28 men, and 20 with vaccinated people, 10 men and 10 women. The socio-demographic characteristics of the people surveyed are shown in Table 2 below.

These results suggest that the profiles of the vaccinated and unvaccinated people we surveyed are very similar. A single trend emerges with regard to gender. Women seem to be more numerous among the unvaccinated. But this would need to be validated statistically.

### Perception of COVID-19 in Benin

We were interested in people’s perception of COVID-19, since acceptance of a vaccine may be linked to their perception of the disease. In the commune of Abomey-Calavi, 65 of the 80 people questioned did not believe in the existence of COVID-19, 90% within unvaccinated people vs 55% within vaccinated people.

All of them said they had not contracted the disease, nor had they seen anyone in their immediate environment contract the disease.

*“Without lying to you, I have my doubts about the reality of this disease. The figures the health authorities are putting forward are just there to scare people... Even when they made a TV film to show the sick at the Allada treatment center, I thought it was a fake”, Maintenance worker, vaccinated, aged 45.*

In addition, among those who did not believe in the existence of the disease, some of them thought that the State made political use of COVID-19 to control political meetings and the movements of its political opponents.

*“Since we’ve been talking about this disease, I’ve never seen anyone around me who’s sick with COVID-19, even the dead, it’s only the governor who sees his dead, laughs...”. Agricultural engineer, unvaccinated, aged 39.*

It should be noted that the start of the epidemic in Benin coincided with a fairly tense electoral period (municipal elections on 17 May 2020). Some members of the public said they had had the impression that restrictions had often been imposed or tightened on the eve of elections. For them, even if the reason officially given was to avoid the spread of the virus, the real intention was quite different.

*“They took these restrictive measures to prevent their opponents from campaigning properly, that’s all. When opponents gather, the police come and disperse them, but when it’s them, nothing happens, it’s the country that’s like that...”, Agricultural engineer, non-vaccinated, 53 years old*

What’s more, some believed that the state was manipulating the test results for political purposes, to restrict the movements of political opponents. A positive result could be attributed to opponents in order to restrict their movements.

*“If this isn’t a set-up, how can someone take their covid PCR test the day before their trip with a negative result in Paris and be PCR positive the next day in Cotonou, there’s something that doesn’t add up...” Mechanic, non-vaccinated, 32 years old*

In addition, 13 of the 80 people interviewed thought that the government was overestimating the number of positive cases in order to obtain more funding from international organizations. In their view, donor funding depended on the number of cases reported. Fifteen respondents (15 out of 80) recognised the existence of COVID-19. However, they relativised the risk they might run, considering that it was a “white man’s disease” that did not affect Africans; Africans were considered to be resistant and to have the necessary immunity to resist the disease.

They also expressed doubts about the seriousness of the disease.

*“This disease can’t kill Africans, otherwise we’d all be dead by now. Look at the people when you’re out and about, there are no barriers, and yet people are doing very well...”, Student, Vaccinated, 20 years old.*

The results of the questionnaires sent to the CHW seem to point in the same direction. Indeed, only 14% of the CHWs were aware of a serious case hospitalised at the epidemic treatment center (ETC) within the population they follow, and this in the two municipalities studied. Deaths appear to be even rarer, with only 2 CHW in Abomey-Calavi reporting a death attributed to COVID-19 in their community and 1 CHW in Zè.

People are therefore surprised by the government’s alarming messages and measures to force vaccination, when the disease is hardly noticeable on a daily basis.

**Fig 1:**
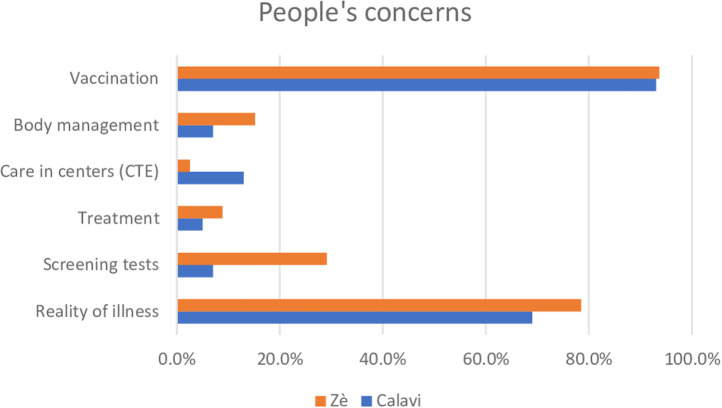
People’s concerns about COVID-19.

Through the testimonies of the CHW, we tried to measure which subjects concerning COVID-19 were the most debated by the population (Fig 1).

Vaccination was the central theme that attracted the attention of over 95% of people in both areas. Similarly, the question of the reality of the disease was raised in the majority of areas managed by CHWs, in both zones. On the other hand, the rural zone is more concerned about the screening tests, their significance and innocuity, and the conditions under which the bodies are managed, whereas in the urban zone, the question of CTE is of concern.

### Vaccination against COVID-19 as a central topic of discussion

Vaccination was the central point of discussion reported by nearly 95% of CHWs in the 2 study areas. Questions asked about vaccination concerned firstly doubts about its efficacy and the fear of post-vaccination death, even 1 or 2 years after vaccination (fear stemming from rumors circulating on social networks). These points were raised also during our qualitative interviews (48 out of 80 people).

These results clearly explain why vaccination was not a priority for the general public. Several reasons were given to justify these doubts. Echoing the information circulating in the media and on social networks, they were concerned firstly that the vaccine had been manufactured in a very short timeframe compared with the WHO’s usual procedures, which take several years. This is reflected in the words of this medical student.

*“How long have we been doing research to find a vaccine against malaria? But up until now, we’ve had nothing really sure, and in a short space of time, we’re told there’s a vaccine against COVID-19. I find that a bit paradoxical...” Student, 25, Abomey-Calavi*

Secondly, they were concerned about vaccines that did not prevent them from contracting the disease, even after they had been injected with full doses. During the focus groups, a CHW shared a statement circulating among the population that they found hard to justify.

*“There’s no point in getting vaccinated if you can still catch the disease...it’s the first time I’ve had to wait for this, it must be because of the haste with which the vaccine was produced.... “ CHW, 32 ans, Abomey-Calavi*

People and CHW also mentioned information that had circulated on social networks, notably concerning side effects following vaccination with AstraZeneca vaccine, which had led to the rejection of the vaccine by the population in the north. This contributed to reinforcing people’s doubts about vaccines, as can be seen from the following verbatim.

*“If even white people complain about side effects, you can see that it’s very serious. In such circumstances, it’s better to avoid this vaccine, or not to get vaccinated at all. I’d rather die from the disease than from the side effects of the vaccine. The after-effects can last a lifetime”, civil servant, 38, Abomey-Calavi.*

Furthermore, some people (1/5) believe that governments are encouraged by technical and financial partners (WHO, UNICEF) not in the goal of protecting the populations of the South, but rather northern populations. In their view, the aim of vaccination in the South is to prevent the virus circulating from the South to the North, where populations are more susceptible. As a result, the funding received from technical and financial partners to combat COVID-19 would also be proportional to the number of people vaccinated.

### Informed consent form for vaccination against COVID-19

Another major point reported was the content of the consent form that people had to read and sign when they were vaccinated. In fact, one of the paragraphs of this form stipulated that the person who had agreed to be vaccinated waived the right to make a complaint against the state or the vaccine manufacturer in the event of a problem (Photo 1: Extract from the informed consent form for vaccination against COVID-19).

For the majority of people interviewed, this passage in the form was an admission that vaccines are unreliable. This paragraph in the consent form made many people reticent and cast suspicion on a state that would not protect its population.

*“I’d gone to be vaccinated of my own free will, so I wasn’t under any pressure. When I got to the health center, the nurse gave me the consent form to read. When I read the part that said the state wasn’t responsible, I was really shocked. I didn’t take any more vaccinations and went home. When I got home, I called one of my cousins in Italy to ask him if it was the same in his country. I realised it wasn’t serious”, Secondary school teacher, unvaccinated, aged 48.*

*“We really had problems with this consent issue. That’s what they were saying all over the village. That’s why a lot of people got discouraged” CHW, Zè*

**Fig 2:**
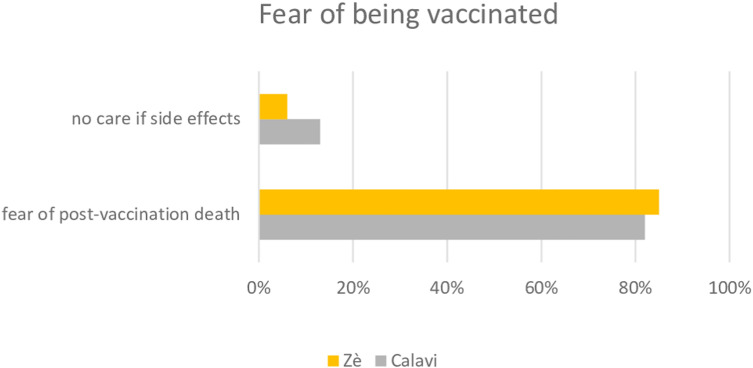
Fear of post vaccination against COVID-19 death.

All these communication errors have resulted in a frightened population, which has ended up being afraid to be vaccinated (Fig 2).

What’s more, these concerns were fueled by the many rumors circulating on social networks.

### Rumours circulating on the social networks about the vaccination

Many rumours circulating on social networks have disorientated people. These were mainly rumours circulating on Whatsapp. These rumours were mainly linked to the risk of post-vaccination deaths and the conspiracy of Northern countries against African countries. In some of these messages, the vaccine was seen as slow-acting poison that could kill between 2 and 5 years after administration.

*“My brother, if you get vaccinated against COVID-19, you’ll die in 2 years, the whites want to kill us. But the God of Africans is great, he will work his miracle...”. Remarks of a housewife relayed by a CHW, Abomey-Calavi*

*“To be vaccinated against hepatitis B, you’re always asked to take a test, otherwise you could die if the virus is in your blood. Now, for COVID-19, they don’t ask you to do any of that and they tell you to go and get vaccinated. Driver, vaccinated, 26, Abomey-Calavi.*

Interviews with local people and CHW showed that people were very focused on an international conspiracy theory. They believed that COVID-19 was intended to make Africans sterile, or even to kill them. The low death rate in Africa was seen as a failure on the part of Northern countries to kill Africans in order to reduce their demographic weight.

For some of those interviewed, vaccination was seen as a second strategy by the countries of the North to achieve their first failed objective. For others, the vaccine itself contained the COVID-19 virus that was to be transmitted to Africans through vaccination.

### Fear of testing positive and body management

The fear of stigmatisation of COVID-19 sufferers and the management of the bodies of people who have died of COVID-19 is a major concern for local people.

For almost half the people questioned, 42 in the population (vaccinated and unvaccinated) and 80 CHW in Abomey-Calavi and Zè, being positive for COVID-19 had serious consequences for individuals. First of all, being quarantined prevented people from working and looking after their families. In a country where 75% of professional activity is informal and very often, daily, quarantine could be synonymous with a loss of income. What’s more, anyone who tested positive was always stigmatised in their environment.

To add to this fear of being recognised as positive, for the people interviewed, being positive meant taking the risk of being sent to a reserved treatment center (CTE), where most deaths occurred. This centralisation of patients, and also of deaths, gave rise in the popular imagination to the idea that these centers (CTEs) were death dens, synonymous with a death sentence if you were admitted there.

*“Have you ever heard of people who have come out of this centre cured? It’s always the dead and always the dead we’re talking about, it’s really frightening”, said a painter relayed by a CHW, Zè.*

Allada is the name of the commune in which the CTE was built to care for the patients of COVID 19. But in Beninese tradition, “going to Allada” has a heavy meaning. Historically, Allada was the capital of a Fon kingdom called Adanwssa (the place of anger, the seat of wrath, in the Fon language). “Going to Allada” is still the expression used today by the Beninese to mean “passing into the afterlife”.

What’s more, in accordance with the rules laid down by the WHO, the rules for managing the bodies of people who died of COVID-19 were extremely strict, depriving the deceased and their families of the necessary rituals in accordance with tradition. These rituals are considered by the local population to be essential to facilitate the deceased’s journey to the afterlife. The consequences of not carrying out these rituals, according to the testimonies gathered, are enormous and can be very serious for the community to which the deceased belongs.

*“There are rituals that must be performed for the deceased, otherwise it’s a curse for generations, illness, accidents, etc. The soul of the deceased will not rest in peace. These rituals are essential”, said a traditional leader relayed by a CHW in Abomey-Calavi.*

*“I’d rather hide and die of COVID-19 than not benefit from the rituals after my death” Doctoral student, unvaccinated, aged 32*

As a result, many people have refused the COVID-19 tests for fear of being found positive and sent to a treatment center.

## Discussion

### Perception of COVID-19 in Benin - The reality and seriousness of the disease

Perception of the disease is one of these factors and our data show that it is particularly important in the case of Benin and COVID-19. The data clearly show that the disease was not perceptible to a large part of the population in Abomey-Calavi in contradiction with government announcements, especially at the start of the epidemic [43]. However, It should be noted that large-scale circulation of the virus in the population of West African countries was only confirmed after serological studies had been carried out, which showed that a majority of the population had been affected by the virus [44–47] but that the vast majority of cases had remained asymptomatic or paucity symptomatic, not warranting a visit to a health center.

At the same time, COVID-19 has been described in many countries, particularly in the North, as a highly contagious, pathogenic and fatal epidemic. Consequently, WHO’s recommendations seemed adapted to northern epidemiological situation but not adapted to southern epidemiological situation and so were poorly accepted by the population.

### Knowledge of the disease and circulation of information

The media were fed questions about vaccination- the speed with which the COVID-19 vaccine was developed, the risk of serious side effects (risk of thrombosis).

In addition, there were errors of communication and implementation during the COVID-19 vaccination campaigns. As a result, the low perception of risk and all the doubts surrounding the vaccine fuelled all the rumours that circulated over the two years and thwarted the vaccination campaigns launched by the WHO and the Beninese government. The results of our study are in line with the work of Wilson 2020 [48], which showed that there is a link between the information circulating on social networks and public doubts about the safety of vaccines. What’s more, the information that has been circulating is similar to what the WHO calls infodemia. An infodemia is an excess of information, including false or misleading information, in the digital and physical environments at the time of the outbreak of a disease. It is a source of confusion and risky behaviour that can damage health. It also leads to distrust of the health authorities and undermines the public health response [49]

A recent article published in Vaccine investigated the factors associated with willingness to be vaccinated against COVID-19 during the pandemic period in Benin [12]. In May 2022, they carried out a quantitative cross-sectional community survey to identify the variables associated with COVID-19 vaccine acceptance.

Acceptance of the vaccine was 43.3%. However, the authors report a proven vaccination rate of 24%, which is a long way from the government’s target of 60% [17]. Neighborhood of residence, level of education, fear of infection, information channel, poor medical conditions, good knowledge of mode of transmission and symptoms, and good behavior were significantly associated with vaccine uptake.

The two studies share some of the factors put forward to explain the relatively low rate of vaccination- afraid of the side effects or doubted effectiveness of the vaccine. They add that some socio-anthropological factors are mentioned such as religious beliefs, cultural practices and attitudes of defiance [12]

Padonou and *al*. add also that there is a difficult context due to false rumours and information that have heightened doubts about vaccination.

### Political use of COVID-19 and vaccination - Vaccination against COVID-19 as a central topic of discussion

Data highlights the importance of the policies followed, the programs proposed and the importance of communication. It has been said, for example, that COVID-19 is being used by African states as a business tool. The funding received from technical and financial partners to fight against COVID-19 would be seen as proportional to the number of cases declared and the number of people vaccinated by the countries. There have also been reports of the political use of COVID-19 to restrict freedom of movement for electoral purposes [50]. Similar findings were also observed in Côte d’Ivoire and Senegal [50,51].

### Response measures and cultural convictions - Fear of testing positive and body management

As part of the measures recommended by the WHO, based on data collected in northern countries to prevent the spread of the epidemic, the sick has been isolated and procedures for managing the bodies have been put in place. These procedures run counter to the mortuary rites of Beninese cultures. The people of Benin have refused to enter a process that could lead the medical profession to declare them dead from COVID-19.

A similar observation was made during the management of the Ebola epidemic in Guinea. Because of the safe burial recommended by the WHO, relatives were not allowed to have access to the bodies for funeral rites. As a result, relatives did not bring the sick to the treatment centers, which amplified the Ebola epidemic. The WHO corrected this by allowing funeral rites to a certain extent, hence the concept of dignified and safe burial to bury the bodies with a degree of dignity [52,53]. Death and the treatment of the deceased, especially in situations where large numbers of people die, seems to be an unresolved issue in epidemic response strategies, probably because biomedical culture combats death more than it manages it [54]. The contribution of the social sciences, and particularly socio-anthropology, to this issue is essential.

### Vaccination consent form not well received

In order to be able to vaccinate against COVID-19, it was compulsory to sign the consent form. Not only is this the very first time that a document of this kind has been introduced into the EPI, but the passage relating to the State’s non-liability in the event of adverse reactions has helped to reinforce doubts about the safety of the vaccine [55,56]. This also contributed to a deterioration in people’s confidence in health professionals and community outreach workers, who are usually considered to be a reliable source.

Discussions with certain authorities at the Ministry of Health and the WHO suggest that the consent form is a WHO recommendation and that its adoption has been the subject of heated debate. However, we note that in the sub-region, only Benin has made this choice, which may have reinforced people’s doubts about the reliability of vaccines.

**Fig 3:**
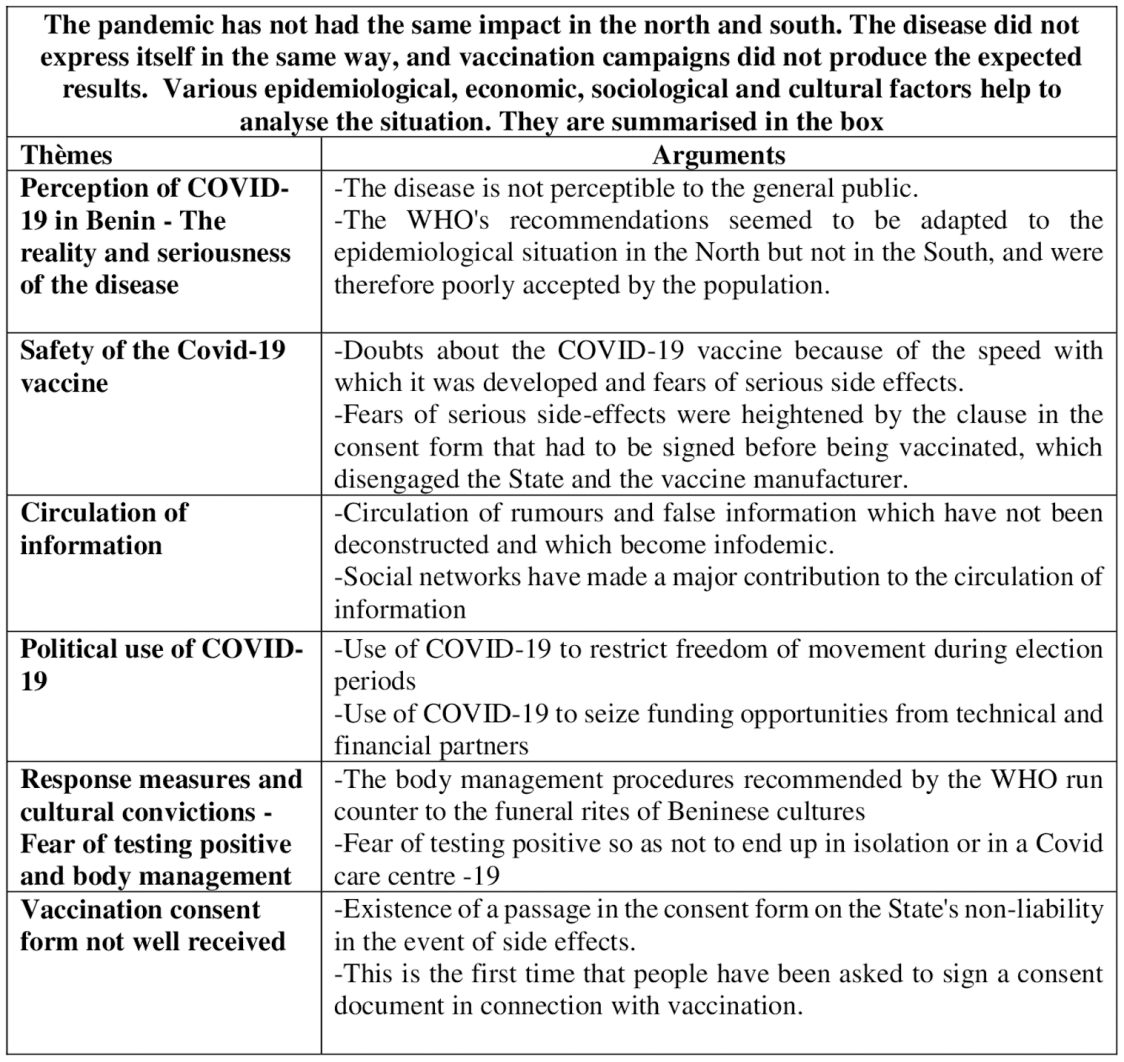
Results box.

The results are summarised in a box (Fig 3).

### Vaccine hesitancy in Benin

The issue of vaccine hesitancy is not specific to the question of anti-COVID-19 vaccination. Even though vaccination campaigns have led to the reduction of many diseases and vaccination is a widely accepted public health measure, particularly for children, the fact remains that certain population groups have strong anti-vaccination convictions [57,58].

The elements of confidence in immunization that emerge from our study are essentially confidence in vaccines - the speed with which vaccines are manufactured and the fear of side effects - confidence in health professionals - the involvement of CHWs in community awareness activities and their defense of the health system - confidence in institutions - perception of the disengagement of the state through the content of the informed consent form, politicized opinion on immunization and the resurgence of conspiracy theories. Trust in institutions - perception of the state’s disengagement through the content of the informed consent form, politicized opinion on vaccination and the resurgence of conspiracy theories.

Eve Dubé’s 2013 article on vaccine hesitancy already proposed a model that evokes the elements of confidence in vaccination. The elements of trust derived from our data are also found in her model [14] as well as in the work of Larson 2018 and Adhikari 2022, which have highlighted and disentangled elements of trust [59,60].

Trust in vaccination arises from interactions between experiences with the healthcare system, different forms of communication and social capital [60,61]. Although in the majority of cases the perceptions and beliefs contributing to trust have commonalities and relevance, trust has often been found to depend on factors rooted in social, cultural, institutional and individual experiences [61]. Understanding the different types of confidence offers ways of encouraging people who are undecided and who refuse to be vaccinated to reconsider their decisions.

## Study strengths and limitations

### Strengths

The data was collected while the epidemic was raging, so that the information circulating among the population could be seen at first hand. The choice of a rural and an urban area enabled a comparison to be made between two broad categories of population living in very different situations in Benin. Finally, the combination of a quantitative and a qualitative approach made it possible first to identify the major issues circulating among the population and then to quantify their extent.

### Limitations

Regarding the qualitative approach, for reasons of time and logistics we were unable to conduct interviews with people in rural areas; however, the quantitative survey of CHW revealed that people’s concerns about COVID-19 and vaccination were not entirely the same in the two areas.

About the quantitative approach, collecting people’s points of view through feedback from CHW certainly enabled us to cover a large study area (the entire commune) and indirectly a large population, but it does not constitute a population-based survey and therefore does not enable us to have a ‘stricto sensu’ representative survey of the general population. However, this approach has made it possible to identify the main questions circulating in the population.

## Conclusion

The COVID19 epidemic took the world by surprise because of its suddenness, the speed with which it spread and the little we knew about the virus and its clinical features. It also surprised the world by the diversity of its clinical manifestations and epidemiological profiles across continents and countries. Countries of the North were the first to be affected, with a very high mortality rate among the elderly. In response, they were the first to institute risk strategies that were often very drastic, population confinement and strong incentives for vaccination. These strategies were transposed to southern countries as the epidemic spread. However, as the manifestations of the disease varied greatly from one area to another, these more or less appropriate responses were often misunderstood or misinterpreted by the population. As a result, the proposed response measures were poorly accepted, starting with vaccination. Like the rest of the world, Africa is connected, and information of all kinds and from all sources has flooded into the population. Errors in communication by national and international authorities fuelled doubts and contributed to the failure of vaccination campaigns.

This widespread hesitancy to vaccinate, which has led to the failure of vaccination campaigns in many countries, should lead us to ask ourselves questions in anticipation of new pandemic episodes that are bound to occur.

Our study, like other studies, clearly shows that any response strategy must take into account the populations of the countries where it is to be applied, with all their socio-cultural, demographic and religious components.

COVID-19 is still present in the world, but less pathological and less lethal. It is no longer the subject of restrictive response measures

The question arises of redefining the COVID-19 vaccination strategy by targeting only so-called at-risk groups. However, it is not certain that these groups are more receptive to being vaccinated. The health authorities consider them to be at risk of serious manifestations of the disease, but there is nothing to suggest that they feel the same way.

Our study confirms the existence of a strong reluctance to vaccinate against COVID-19 in the general population and shows the absolute necessity for the authorities to take these different messages into account in their communications if they decide to relaunch vaccination, whether on a mass scale or aimed at risk populations.

## Supporting information

S1 Text Questionnaire. (DOCX)

S2 Text Interview guide. (DOCX)
