# Supplementary material for: Understanding the vaccine hesitancy of COVID-19 in Benin
Source: PLOS Glob Public Health. 2025 Feb 25;5(2):e0004267. doi: 10.1371/journal.pgph.0004267 (PMC11856262; doi:10.1371/journal.pgph.0004267)
Supplement: S1 Text — (DOCX) [file pgph.0004267.s001.docx]

**Enquête STRESSCO chez les relais communautaires sur**

**l'hésitation vaccinale**

## Questionnaire relais communautaire

| Questions | Modalités |
| --- | --- |
| \|  \| **IDENTIFICATION** \| \| --- \| --- \| | |
| Age | ---- |
| Sexe | -M  - F |
| Niveau d’instruction | - Aucun - Primaire - Secondaire - Universitaire |
| Situation matrimoniale | - Célibataire - Marié - Divorcé - Vie en couple |
| Activités socio-professionnelles |  |
| Appartenance religieuse : | - Catholique - Protestant - Musulman - Adventiste - Evangélique - Religion endogène - Autres ........... |
| Statut du relais communautaire : | - Paquet complet - Paquet promotionnel - Autres ............................ |
| Statut salarial : | - ONG - MS - Bénévolat - Autres.............................. |
| % de temps consacré : | - 0-20% - 20- 50% - 50- 70% - 70- 100% |

|  | | |  |
| --- | --- | --- | --- |
| \|  \| **TEST COVID** \| \| --- \| --- \| | | | |
| Questions | | Modalités | |
| Avez-vous déjà réalisé un test COVID ? | - Oui - Non | | |
| Si non, pour quelle raison ? | - Pas de signe suspect - Pas de contact avec un cas suspect ou positif - Pas confiance au test - Peur d’être testé positif - Autres | | |
| Connaissez vous des personnes qui refusent de se faire dépister ? | - Oui - Non | | |
| Selon vous, pourquoi elles refusent le dépistage? | - Ne sont pas informées - Coût du dépistage - Stigmatisation - Mauvais accueil - Peur d’être testé positif - Pas confiance au test - Ne sait pas - Autres | | |

| \|  \| **FORMATION/ RESPONSABILITE** \| \| --- \| --- \| | |
| --- | --- | --- | --- |
| Avez-vous reçu des formations sur le Covid et la vaccination ? | - Oui - Non |
| Si oui, combien de fois avez-vous suivi une formation ? | -------- |
| La dernière formation a été organisée par qui ? | -------- |
| Comment appréciez-vous le déroulement de la formation ?  NB : Choisir une modalité et justifier | - Mauvais - Acceptable - Bien |
| En tant que relais Communautaire (RC) quelle tâche vous a été assignée ? (plusieurs cases peuvent être cochées) | - Sensibilisation - Détection de cas - Dépistage - Traitement - Amener les populations au centre de vaccination - Autres |
| Avez-vous eu connaissance de cas grave, de décès dû au COVID-19 dans votre communauté? | - Oui - Non |
| Si oui, combien de cas grave  ? | - Inférieur à 5 - Entre 5 et 10 - Supérieur à 10 |
| Si oui quelle, combien de décès ? | - 0 - Entre à 5 - Entre 5 et 10 - Supérieur à 10 |

| \|  \| **PERCEPTION DU VACCIN PAR LE RELAIS** \| \| --- \| --- \| | |
| --- | --- | --- | --- |
| Etes-vous vacciné ? | - Oui - Non |
| Si oui, dans quelle circonstance vous vous êtes-vous fait vacciner ? | - Volontaire (sans sensibilisation) - Volontaire (après sensibilisation) - Contrainte professionnelle - Contrainte administrative - Autres |
| Avez-vous passé l’information en communauté comme quoi vous vous êtes déjà vacciné ? | -Oui  - Non |
| Si non, pourquoi | - Peur d’être envouté - Ne veut pas être qualifié de peureux - Ne veut pas alarmer son entourage - Peur d’être objet polémique - autres |
| Si oui, pourquoi ? | - Convaincre un parent proche - Convaincre les populations - Autres   ----- |
| Savez-vous des effets indésirables ou secondaires liés à la vaccination? | - Oui - Non |
| Si oui, citez 3 effets indésirables ou secondaires que vous redoutez le plus sur les vaccins contre la COVID 19 ? | -1) -------2) _____3)_____ |
| Avez-vous eu connaissance d'effets indésirables après la vaccination anti COVID-19 dans votre communauté? | - Oui - Non |
| Si oui, lesquels ?  NB : (plusieurs cases peuvent être cochées) | - Fièvres - Douleur - -fatigue - Lourdeur - sommeil - Perte de l’immunité - Mort - Paralysie momentané - Impuissante sexuelle momentané - Autres |
| Ces personnes ont-elles été prises en charge par des agents de santé ? | - Oui - Non |
| Si oui, est-ce gratuitement ? | - Oui - Non |

| \|  \| **PERCEPTION DU COVID et du VACCIN PAR LES POPULATIONS** \| \| --- \| --- \| | |
| --- | --- | --- | --- |
|  |  |
| Quelles sont les sujets que les populations abordent le plus avec vous ?  NB : (plusieurs cases peuvent être cochées) | - Réalité de la maladie - Test de dépistage - Traitement - Prise en charge CTE - Gestion des corps - Vaccination - autres |
| D'après vous qu’est ce qui fait que la population hésite à se faire vacciner ?  NB : (plusieurs cases peuvent être cochées) | - Peur de ne pas être pris en charge en cas d’effets secondaire - Clauses de non recours à la justice - Peur de la mort - Autres |
| Avez vous fait face à des refus absolu de se vacciner? | - rarement - parfois - souvent - tres souvent |

| \|  \| **DECISION DU GOUVERNEMENT** \| \| --- \| --- \| | |
| --- | --- | --- | --- |
| Le gouvernement a pris des décrets pour amener directement ou indirectement certains corps de métier ou catégories de personne à se faire vacciner. Selon vous, ces mesures sont comment ? | - Bonne - Mauvaise - Ne sait pas |
| Que pensent les gens en communauté de ces décisions du gouvernement ? | - Bonne - Mauvaise - Ne sait pas |
| Que pensez-vous de la mise en place des comités d’engagement covid ? | - Bonne - Mauvaise - Ne sait pas |
| Que pensez-vous du fonctionnement des comités d’engagement covid ? | - Bonne - Mauvaise - Ne sait pas |

| \|  \| **ACCESSIBILITE DE LA VACCINATION POUR LES POPULATIONS** \| \| --- \| --- \| | |
| --- | --- | --- | --- |
| Pensez-vous qu’il est facile de se faire vacciner dans votre localité ? | - Oui - Non |
| Si non pourquoi ?  NB : (plusieurs cases peuvent être cochées) | - Distance - Rupture de vaccin préféré - Mauvaise information sur le vaccin - Manque d’information sur les lieux de vaccination - Mauvais accueil des agents de santé - Autres |

| \|  \| **VACCIN CHEZ LES ENFANTS** \| \| --- \| --- \| | |
| --- | --- | --- | --- |
| Pensez-vous qu'il faille vacciner les enfants entre 12 et 18 ans ? | - Oui - Non |
| Si non, pourquoi ? | - Risque élevé pour les enfants - Enfants naturellement immunisés - Autres |
|  |  |

| \|  \| **Confiance dans les autorités pour informer sur les vaccins** \| \| --- \| --- \| | |
| --- | --- | --- | --- |
| Je fais confiance au ministère la santé pour fournir des informations fiables sur les risques et les avantages des vaccins | Echelle de 1 à 10 |
| Je fais confiance à la science pour développer de nouveaux vaccins sûrs et efficaces* | Echelle de 1 à 10 |
| Je vais confiance au ministère de la santé pour garantir des vaccins sûrs | Echelle de 1 à 10 |
| Je suis convaincu que le MS ne se laisse pas influencé par la pression des organisation internationale (OMS, les industrie pharmaceutique, gouvernement du Nord) | Echelle de 1 à 10 |

| **Ouverture aux préoccupations des patients concernant la vaccination** | |
| --- | --- |
| Est-ce que vous vous retrouvez dans cette affirmation  « J’essaye de convaincre ma communauté sur les avantages et les risques des vaccins mais je les laisse prendre leur décision sans essayer de les influencer » | - Oui - Non |
| Est-ce que vous vous êtes d’accord avec cette affirmation : « Les populations ont le devoir de se faire vacciner » | - Oui - Non |
| Est-ce que vous vous retrouvez dans cette affirmation  « Je suis prêt à laisser les parents retarder la vaccination des adolescents (12 à 18 ans) » | - Oui - Non |

| **Auto-efficacité dans la lutte contre l’hésitation** | |
| --- | --- |
| Je me sens à l’aise pour discuter des vaccins avec les membres de ma communauté qui hésitent fortement à se faire vacciner  Echelle ? | Echelle de 1 à 10 |
| Je me sens suffisamment informer et former pour discuter des vaccins avec les populations | Echelle de 1 à 10 |
