# Supplementary material for: Understanding the vaccine hesitancy of COVID-19 in Benin
Source: PLOS Glob Public Health. 2025 Feb 25;5(2):e0004267. doi: 10.1371/journal.pgph.0004267 (PMC11856262; doi:10.1371/journal.pgph.0004267)
Supplement: S2 Text — (DOCX) [file pgph.0004267.s002.docx]

## Guide d’entretien enquête pour leS vaccinés

**1. Contexte de déroulement de l’entretien -nom de l'interviewer**

- Date :
- Lieu :
- Heure de début :
- Heure de fin :
- Circonstance de l’entretien :

**2. Identification**

- Nom et prénoms : (A demander à la fin, mettre dans le carnet de terrain)
- Age :
- Sexe :
- Niveau d’instruction :
- Situation matrimoniale (si en couple - quelques infos sur le-la conjoint (e) (niveau instruction, profession)
- Activité(s) socioprofessionnelle(s) :
- Groupe sociolinguistique :
- Appartenance religieuse :
- Niveau socio –économique : (location ou propriétaire de logement ? Possède moto ou voiture ou rien ?

***Contexte / cadre de vie***

Avez-vous vécu une expérience COVID

1) personnellement (infection ?) (Vécu de la maladie, test de dépistage, prise en charge)Décrire en détail

2) avec votre entourage à propos du COVID 19 (infection ?) (Vécu de la maladie, test de dépistage, prise en charge, décès (gestion des corps?) Décrire en détail.

Avez-vous déjà fait un test COVID ? Si oui, pour quelle raison ? Comment appréciez-vous ce test ?

Si non - jamais l'occasion ?

Ou : avez-vous déjà refusé de faire un test Covid ? Pourquoi ?

Selon vous, pourquoi certaines personnes n’aiment pas se faire dépister ?

***Informations sur le Covid 19 / Infodémie***

Que savez- vous de la COVID 19 ; sur son origine; son existence ( réalité de la maladie ou non ?), son mode de transmission ?, la prévention et le traitement?

Que pensent les gens de votre entourage du COVID 19.

Quelles sont les informations qui circulent sur les réseaux sociaux à propos du COVID-19 ? (Test de dépistage, traitement, pris en charge CTE, vaccination, gestion des corps, etc.).

Quelles sont vos sources d’informations ?

Que pensez-vous de ces informations? Vous rassurent-elles ? vous inquiètent-elles ? Pourquoi ?

Pensez-vous être suffisamment informé sur le COVID 19 ? Pourquoi ?

Pensez-vous que le gouvernement communique assez sur la Covid ? Si non, pourquoi?

***Informations sur le Vaccin***

Vous vous êtes fait vacciner - pour quelles raisons ? (quels ont été vos motivations? dans quelles conditions ?)

Pourquoi avez-vous décidé de vous faire vacciner malgré toutes les rumeurs qui circulent à propos des vaccins contre la COVID 19 ?

Que savez-vous du vaccin?

Quelles sont vos sources d’informations sur le vaccin ?

Pensez-vous être suffisamment informé sur le vaccin ?

Que pensez-vous de la communication qui est faite sur le vaccin ? Qu’avez-vous retenu de la multitude d’informations qui circulent sur le vaccin.

***Accessibilité et acceptabilité du vaccin contre la COVID-19***

Connaissez-vous les vaccins qui sont proposées actuellement dans les centres de santé ? Que pensez-vous de ces vaccins ? Connaissez-vous d’autres vaccins ? Qu’en pensez-vous ? (Pour mémo, les vaccins disponibles sont Sinovac, Johson-Johson, Atra Zénéka, Pfizer)

Quel vaccin avez-vous fait ? Quels ont été les critères de votre choix ? Si ce vaccin n’était pas disponible, quelle serait votre attitude ?

Le gouvernement a pris des décrets pour amener directement ou indirectement certains corps de métier ou catégories de personne à se faire vacciner (les agents de santé, les militaires, les enseignants, les gardes malade), que pensez-vous de cette décision du gouvernement ?

Il se pourrait qu’on demande à ceux qui se sont déjà vaccinés complètement de faire une dose supplémentaire pour une bonne immunité ? Qu’est-ce que cela vous inspire ? Accepteriez-vous de faire une dose supplémentaire pour renforcer votre immunité ? Pourquoi ?

A-t-il été facile pour vous de vous faire vacciner ? (Accessibilité au centre de vaccination, temps d’attente, disponibilité du vaccin, accueil des agents, etc.).

***Efficacité perçues et acceptabilité du vaccin contre la COVID-19***

Que pensez-vous de ceux qui pensent que les vaccins contre la COVID-19 ne sont pas sécurisants ? Etes-vous du même avis ? Pourquoi ?

Quels sont les effets indésirables ou secondaires que vous redoutez le plus sur les vaccins contre la COVID 19 ? Expliquez.

Avez-vous eu connaissance des effets après la vaccination contre la COVID-19 ? Si oui, lesquels ?? Quelles ont été vos sources d’informations ? (media?, personne proche?, source médicale?

Vous a-t-on déconseillé de vous faire vacciner ? Qui ? si oui, pourquoi malgré cela vous vous êtes vacciné ?

En évaluant le bénéfice/ risque dans le contexte Béninois, pensez qu’il serait judicieux que les gens se vaccinent ? Pourquoi ?

Quelles recommandations faites-vous pour une adhésion des populations à la vaccination ?

Actuellement, un nouveau mode d'administration des vaccins par voie nasale est en expérimentation. Si ces vaccins arrivent au Bénin, seriez-vous prêts les gens seront prêt à se faire vacciner ? Pourquoi?

**Merci ! issue**

## Guide d’entretien enquête pour les non vaccinés

**1. Contexte de déroulement de l’entretien -nom de l'interviewer**

- Date :
- Lieu :
- Heure de début :
- Heure de fin :
- Circonstance de l’entretien :

**2. Identification**

- Nom et prénoms : (A demander à la fin, mettre dans le carnet de terrain)
- Age :
- Sexe :
- Niveau d’instruction :
- Situation matrimoniale (si en couple - quelques infos sur le-la conjoint (e) (niveau instruction, profession)
- Activité(s) socioprofessionnelle(s) :
- Groupe sociolinguistique :
- Appartenance religieuse :
- Niveau socio –économique : (location ou propriétaire de logement ? Possède moto ou voiture ou rien ?

***Contexte / cadre de vie***

Avez-vous vécu une expérience COVID

1) personnellement (infection ?) (Vécu de la maladie, test de dépistage, prise en charge)Décrire en détail

2) avec votre entourage à propos du COVID 19 (infection ?) (Vécu de la maladie, test de dépistage, prise en charge, décès (gestion des corps?) Décrire en détail.

Avez-vous déjà fait un test COVID ? Si oui, pour quelle raison ? Comment appréciez-vous ce test ?

Si non - jamais l'occasion ?

Ou : avez-vous déjà refusé de faire un test Covid ? Pourquoi ?

Selon vous, pourquoi certaines personnes n’aiment pas se faire dépister ?

***Informations sur le Covid 19 / Infodémie***

Que savez- vous de la COVID 19 ; sur son origine; son existence ( réalité de la maladie ou non ?), son mode de transmission ?, la prévention et le traitement?

Que pensent les gens de votre entourage du COVID 19.

Quelles sont les informations qui circulent sur les réseaux sociaux à propos du COVID-19 ? (Test de dépistage, traitement, pris en charge CTE, vaccination, gestion des corps, etc.).

Quelles sont vos sources d’informations ?

Que pensez-vous de ces informations? Vous rassurent-elles ? vous inquiètent-elles ? Pourquoi ?

Pensez-vous être suffisamment informé sur le COVID 19 ? Pourquoi ?

Pensez-vous que le gouvernement communique assez sur la Covid ? Si non, pourquoi?

***Informations sur le Vaccin***

Vous n'êtes pas vacciné - pour quelles raisons ?

Que savez-vous du vaccin?

Quelles sont vos sources d’informations sur le vaccin ?

Pensez-vous être suffisamment informé sur le vaccin ?

Que pensez-vous de la communication qui est faite sur le vaccin ? Qu’avez-vous retenu de la multitude d’informations qui circulent sur le vaccin.

***Accessibilité et acceptabilité du vaccin contre la COVID-19***

Savez-vous où l'on peut se faire vacciner?

Comment avez-vous eu l’information ?

Connaissez-vous les vaccins qui sont proposées actuellement dans les centres de santé ?

Que pensez-vous de ces vaccins ?

Connaissez-vous d’autres vaccins ? Qu’en pensez-vous ? (Pour mémo, les vaccins disponibles sont Sinovac, Johson-Johson, Atra Zénéka, Pfizer)

Si vous devriez vous vacciner, quel vaccin préféreriez-vous ? Pourquoi ? Si ce vaccin n’est pas disponible, quel sera votre attitude ?

Le gouvernement a pris des décrets pour amener directement ou indirectement certains corps de métier ou catégories de personne à se faire vacciner (les agents de santé, les militaires, les enseignants, les gardes malade), que pensez-vous de cette décision du gouvernement ?

Il se pourrait qu’on demande à ceux qui se sont déjà vaccinés complètement de faire une dose supplémentaire pour une bonne immunité ? Qu’est-ce que cela vous inspire ?

Pensez-vous qu’il est facile de se faire vacciner (accessibilité au centre de vaccination, temps d’attente, disponibilité du vaccin, accueil des agents de santé, etc.)

***Efficacité perçues et acceptabilité du vaccin contre la COVID-19***

Que pensez-vous de ceux qui pensent que les vaccins contre la COVID-19 ne sont pas sécurisants ? Etes-vous du même avis ? Pourquoi ?

Quels sont les effets indésirables ou secondaires que vous redoutez le plus sur les vaccins contre la COVID 19 ? Expliquez.

Avez-vous eu connaissance des effets après la vaccination contre la COVID-19 ? Si oui, lesquels ?? Quelles ont été vos sources d’informations ? (media?, personne proche?, source médicale?

Vous a-t-on déconseillé de vous faire vacciner ? Qui ? Si oui, pensez-vous que cela a été déterminant dans votre choix de ne pas vous faire vacciner ?

En évaluant le bénéfice/ risque dans le contexte Béninois, pensez qu’il serait judicieux que les gens se vaccinent ? Pourquoi ?

A quelles conditions accepteriez-vous de vous faire vacciner ?

Quelles recommandations faites-vous pour une adhésion des populations à la vaccination ?

Actuellement, un nouveau mode d'administration des vaccins par voie nasale est en expérimentation. Si ces vaccins arrivent au Bénin, seriez-vous prêt à vous faire vacciner ? Pourquoi ?

**Merci !**

**Guide d’entretien pour les relais communautaires sur**

**l'hésitation vaccinale**

**1. Contexte de déroulement de l’entretien -nom de l'interviewer**

- Date :
- Lieu :
- Durée de l’entretien

**2. Identification**

- Age :
- Sexe :
- Niveau d’instruction :
- Situation matrimoniale (si en couple - quelques infos sur le-la conjoint (e)
- Activité(s) socioprofessionnelle(s) :
- Groupe sociolinguistique :
- Appartenance religieuse :
- Statut du relais communautaire :
- % de temps consacré
- Téléphone

***Contexte / cadre de vie***

Avez-vous vécu une expérience COVID

1) personnellement (infection ?) (Vécu de la maladie, accessibilité du test de dépistage (gratuit ou payant), prise en charge (prise en charge personnel ou centre de santé) Décrire en détail

2) avec votre entourage à propos du COVID 19 (infection ?) (Vécu de la maladie, accessibilité du test de dépistage, prise en charge, décès (gestion des corps ?) Décrire en détail.

Avez-vous déjà fait un test COVID ? Si oui, pour quelle raison ? Comment appréciez-vous ce test ?

Avez-vous déjà refusé de faire un test Covid ? Pourquoi ?

Selon vous, pourquoi certaines personnes n’aiment pas se faire dépister dans votre communauté ?

***Informations sur le Covid 19 / Infodémie***

Que savez- vous de la COVID 19 ; sur son origine ; son existence (réalité de la maladie ou non ?), son mode de transmission ? la prévention et le traitement ?

Avez-vous reçu des formations sur la Covid et sa prise en charge ? Combien ? Comment la formation s’est-elle déroulée ? Qui étaient les organisateurs ? Durée ? Cette formation répondait –elle à vos attentes ? Pourquoi ?

Pensez-vous être suffisamment informé sur la COVID 19 ? Pourquoi ?

Quelle tâche vous a été assignée ? informer, diriger, traiter, dépister ?

Pensez-vous que dans le centre de santé où vous travaillez sensibilise suffisamment sur la COVID 19 ? Si non, pourquoi ? Si oui pourquoi ?

***Informations sur le Vaccin***

Que pensez-vous du vaccin COVID 19 comparativement aux autres vaccins du PEV ?

Avez-vous reçu des formations sur le vaccin et la vaccination ? Si oui, comment la formation s’est-elle déroulée ? Qui étaient les organisateurs ? Cette formation répondait –elle à vos attentes ? Pourquoi ?

Pensez-vous être suffisamment informé sur le vaccin ? Oui/ non Pourquoi ?

Que pensez-vous de la communication qui est faite sur le vaccin ? Qu’avez-vous retenu de la multitude d’informations qui circulent sur le vaccin.

Quelle tâche vous a été assignée ? Informer, diriger vers la vaccination ?

***Perception du vaccin par le relais***

Etes-vous vacciné ?

Si oui, pouvez-vous nous expliquer dans quelle circonstance vous vous êtes vacciné ? action volontaire, contrainte professionnelle. Comment avez-vous vécu cela ?

Pensez-vous qu'il y a des effets secondaires à la vaccination ? oui/ non, pourquoi ?

Quelle est votre perception du vaccin ? Que pensez-vous personnellement de chacun de ces vaccins ( efficacité, rappel), ? (Pour mémo, les vaccins disponibles sont Sinovac, Johson-Johson, Atra Zénéka, Pfizer, spoutnik, moderna)

***Perception du vaccin par la population***

D'après vous quelles sont les craintes de la population vis à vis du vaccin ?

Par exemple sur la clause de non-recours à la justice

Par exemple les effets secondaires du vaccin

Que pensez-vous de ces craintes ?

Avez-vous passé l’information comme quoi vous vous êtes déjà vacciné en communauté ? Si non pourquoi ? Si oui, quelle a été leur attitude ?

Pensez-vous qu'il existe une hostilité de la population envers vous dû à votre rôle pour la mobilisation pour la vaccination ? pourquoi ?

Pensez-vous que c’est une tâche facile ou compliquée pour vous d’amener les gens se faire vacciner ? Expliquez

**Action du gouvernement**

Le gouvernement a pris des décrets pour amener directement ou indirectement certains corps de métier ou catégories de personne à se faire vacciner (les agents de santé, les militaires, les enseignants, les gardes malade),

Que pensez-vous de cette décision du gouvernement ?

Que pensent les gens en communauté de ces décisions du gouvernement ?

Que pensez-vous de la création et du fonctionnement des comités d’engagements Covid ?

**Accessibilité de la vaccination pour les populations**

Pensez-vous qu’il est facile de se faire vacciner dans votre localité (accessibilité au centre de vaccination, temps d’attente, disponibilité du vaccin, accueil des agents de santé, etc.) ? Recevez-vous des plaintes dans ce sens ? que disent les gens ?

Avez-vous eu connaissance des effets secondaires ou indésirables après la vaccination contre la COVID-19 dans votre communauté ? Si oui, lesquels ? Comment cela a-t-il été géré ?

Que pensez-vous de ceux qui pensent que les vaccins contre la COVID-19 ne sont pas sécurisants ? Etes-vous du même avis ? Pourquoi ?

Quels sont les effets indésirables ou secondaires que vous redoutez le plus sur les vaccins contre la COVID 19 ? Expliquez.

En évaluant le bénéfice/ risque dans le contexte Béninois, pensez qu’il serait judicieux que les gens se vaccinent ? Pourquoi ?

Que pensez-vous du déroulement des différentes campagnes de vaccination ? Qu’est-ce qui vous a marqué positivement ? Qu’est-ce qui vous a marqué négativement ?

Que pensez vous de l’arrêt des campagnes de vaccinations ?

**Vaccin chez les enfants**

Pensez-vous qu'il faut vacciner les enfants entre 12 et 18 ans ? Expliquez votre réponse

Les parents sont-ils favorables à la vaccination des enfants dans votre localité ? Pourquoi ? Donnez des exemples

**Discussion avec le relais sur son propre engagement**

**Confiance dans les autorités pour informer sur les vaccins**

Avez-vous confiance à l’état ou au ministère de la santé pour ce qui est de l’efficacité et de la sécurité des vaccins ? expliquez votre réponse ?

Avez-vous confiance à la science ou aux firmes pharmaceutique pour ce qui est de la sécurité et l’efficacité des vaccins ? expliquez votre réponse ?

Avez-vous confiance aux informations que le ministère fait passer sur la vaccination Covid ? si oui pourquoi ? si non pourquoi ?

**Ouverture aux préoccupations des populations concernant la vaccination**

Pensez-vous que les personnes qui hésitent sur les avantages et les risques des vaccins ont des raisons légitimes ? expliquez votre réponse ?

Pensez-vous qu’il est contreproductif d’essayer de convaincre les personnes réfractaires à la vaccination contre la Covid 19 ? Expliquez votre réponse

Pensez-vous que les populations ont le devoir de se faire vacciner ? Expliquez votre réponse

**Auto-efficacité dans la lutte contre l’hésitation**

Sentez-vous suffisamment informer et former pour discuter des vaccins avec **tous** les membres de votre communauté ? Expliquez votre réponse

Sentez-vous à l’aise pour discuter des vaccins avec les membres de votre communauté qui hésitent fortement à se faire vacciner ? Expliquez votre réponse

Quelles recommandations faites-vous pour une adhésion massive des populations à la vaccination ?

Merci
